# Clownfish in hypoxic anemones replenish host O_2_ at only localised scales

**DOI:** 10.1038/s41598-017-06695-x

**Published:** 2017-07-26

**Authors:** N. A. Herbert, S. Bröhl, K. Springer, A. Kunzmann

**Affiliations:** 1 Leigh Marine Laboratory, Institute of Marine Science, University of Auckland, PO Box 349, Warkworth, 0941 New Zealand; 2 Leibniz Center for Tropical Marine Research (ZMT), Fahrenheitstraße 6, 28359 Bremen, Germany; 3 Marine Botany BreMarE, University of Bremen, Leobenerstr.1, 28359 Bremen, Germany

## Abstract

The clownfish-anemone association exemplifies a symbiosis where both members benefit from nutrient exchange and protection from predators. Clownfish also perform aeration-like behaviour in their host anemones at night, but it is not yet known whether this is stimulated by the onset of hypoxia, and whether both members benefit from O_2_ replenishment. Oxygen at 3 distances above the sea anemone *Entacmaea quadricolor* (0.2, 1.2 and 2.2 cm) therefore was measured under 3 light levels (photon flux density = 0, 55 and 110 µmol m^−2^ s^−1^), with and without the anemonefish *Amphiprion frenatus*. Hypoxia (O_2_ < 50% air saturation) was recorded in the anemone, but only at 0.2 cm away from the anemone surface under dark conditions when *A. frenatus* was absent. This localised layer of hypoxia was eliminated by the presence of *A. frenatus* exhibiting aeration-like behaviour. Respirometry revealed that *A. frenatus* is extremely hypoxia tolerant (*S*_crit_ = 14.3% at 25 °C), suggesting that aeration behaviour does not provide a major metabolic advantage to clownfish because they do not breathe water at 0.2 cm and are not metabolically constrained by O_2_ at distances ≥ 1.2 cm. That the aeration behaviour of *A. frenatus* facilitates only the metabolism of its O_2_-conforming host reveals a unique aspect of this symbiotic relationship.

## Introduction

The relationship between clownfishes and sea anemones is a highly popularised example of symbiosis, in which both members confer a mutual benefit to each other in terms of protection from predators^1, 2^ and the exchange of food and nutrients^1, 3, 4^. The relationship between clownfishes and anemones is apparently obligate, because clownfish are readily predated beyond the protective range of the anemone^1^ and anemones do not thrive in terms of growth and reproduction without clownfish^2, 5^. Szczebak *et al*.^6^ also recently have added an interesting behavioural and physiological dimension to this association, showing that clownfish undertake flow-modulating (i.e. aeration-like) behaviour in anemones at night, and that physical contact raises the O_2_ consumption rate of the combined pairing. Whether it was the clownfish and/or the anemone that contributed to the increase in O_2_ consumption could not be resolved, but it was concluded that the flow modulating behaviour of clownfish probably oxygenates the anemone and thus acts to augment metabolism, possibly in both partners. However, neither Szczebak *et al*.^6^, nor indeed anyone else to our knowledge, has provided measures of O_2_ in and around the anemone, so it is not yet known whether the night time aeration-like behaviour of clownfish does actually oxygenate the anemone, and whether an environmental cue such as hypoxia serves to stimulate this behaviour. On that basis, it is also not yet clear whether metabolic respiratory advantages in the clownfish-anemone association are shared by both parties, and thus contribute to 2-way mutualistic benefits in this symbiosis.

There is a paucity of information regarding O_2_ in and around the tentacles of sea anemones, but data exist regarding O_2_ in and around the polyps of hermatypic reef corals^7–9^. Because some anemones possess photosynthetic zooxanthellae (e.g. the bulb tentacle anemone, *Entacmaea quadricolor*) and therefore share similar features with coral polyps, the published literature regarding the O_2_ environment and association of fish around photosynthesising corals may provide a useful basis for hypotheses concerning the tripartite relationship between clownfishes, sea anemones and resident zooxanthellae. When examining O_2_ in and around the branches of tropical corals, large diel O_2_ fluctuations have been observed and appear to reflect the balance between respiratory O_2_ consumption of animal tissues and the O_2_ yield of photosynthetic zooxanthellae during daylight hours. Indeed, the O_2_ environment above the surface of live hermatypic corals is reportedly hyperoxic by day (O_2_ > 300% air saturation) and severely hypoxic or even anoxic at night (O_2_ < 10% air saturation)^8, 9^. However, for the purposes of the current study, it is important to establish whether the magnitude of the diel hypoxic-hyperoxic cycle is contained with the boundary layer of cnidarians or extends a relevant distance away from the polyp surface, where clownfish might also experience cycles of hyperoxia and hypoxia. Any insight into the influence of fish presence on these O_2_ profiles would also of course be extremely relevant to the study conclusions of Szczebak *et al*.^6^ and the hypotheses of the current study. In that respect, the studies of Shashar *et al*.^9^ and Kühl *et al*.^8^ provide vital information, because the diel cycle in O_2_ appears to diminish rapidly with only a small increase in distance from the polyp surface. Using the branching coral *Stylophora pistillata* as an example, hypoxia at night and hyperoxia during the day remains measurable only across a very narrow 3 mm boundary layer in calm water, and even less in water with a 5 cm s^−1^ current^9^. Nilsson and Östlund-Nilsson^10^ and Nilsson *et al*.^11^ report O_2_ levels below 20% of air saturation between the branches of corals, but unfortunately did not provide details regarding the exact distances of these readings above the coral tissue surface and it is likely that water flow was negligible in these coral branches. It is not therefore clear whether these O_2_ readings relate specifically to the narrow boundary layer above corals, or to water in the areas where symbiotic fish are likely to breathe.

The data of Goldshmid *et al*.^7^ confirm the existence of severely hypoxic water above the surface of the branching coral *S. pistallata* at night, and more importantly demonstrate that resident damselfish *Dascyllus marginatus* increase O_2_ to a non-hypoxic level (i.e. >50% air saturation). However, the O_2_ measures of Goldshmid *et al*.^7^ were made at a distance of only 1 mm from the coral surface, so their O_2_ data simply confirm the observations of Shashar *et al*.^9^ and do not support the idea of hypoxia being widespread in coral branches. Therefore, given the body of evidence to date, it is a distinct possibility that hypoxia and/or hyperoxia is physiologically relevant only to corals and not to the fish that reside among their branches. If the same pattern holds true for the clownfish-anemone association, we hypothesize that any significant change in O_2_ (whether it be hyperoxia and/or hypoxia) would also be present and modulated by fish at a scale of only a few millimetres above the anemone. However, whilst the above focuses on the potential constraints of low O_2_ (hypoxia), the flow-modulating effects of fish on hyperoxia should not be ignored, because the pulsating movement of soft corals is known to enhance the rate of photosynthesis of associated zooxanthellae^12^. This is because pulsation increases water flow and provides a net efflux of excess O_2_, which subsequently facilitates the binding affinity of RuBisCo for CO_2_^12^. In this situation, the movement of symbiotic fish in and out of sea anemones by day could also potentially enhance the photosynthetic flow of energy in this symbiosis by encouraging water movements that minimise the extent of hyperoxia during daylight hours.

The tomato clownfish, *Amphiprion frenatus*, and the bulb tentacle anemone, *Entacmaea quadricolor*, are employed here as a model symbiosis to test the following set of questions and hypotheses: 1) How does O_2_ level vary with distance from the surface of *E. quadricolor*, and how does light (hence the balance between respiration and photosynthesis) influence the O_2_ profiles observed? For this particular question, information in Shashar *et al*.^9^ and Kühl *et al*.^8^ was used to formulate the specific hypothesis that both hyperoxia and hypoxia will be observed, but only at measurement scales relevant to sea anemones (i.e. a distance of a few millimetres, not centimeters, above *E. quadricolor*). 2) Does the presence of *A. frenatus* modulate the O_2_ profiles observed in *E. quadricolor*? Here, it is specifically hypothesized that the aeration-like (i.e. flow modulating) behaviour of clownfish^6^ has the potential to reduce hyperoxia under illuminated conditions as well as hypoxia under dark conditions. 3) Finally, based on the results of 1 and 2 above, intermittent flow respirometry was used to establish whether *A. frenatus* can physiologically tolerate the O_2_ levels observed among the tentacles of sea anemones. This will allow us to ascertain whether the aeration-like (i.e. flow modulating) behaviour of clownfish^6^ is potentially activated in response to low O_2_ intolerance and/or whether clownfish are likely to benefit metabolically from their aeration-like behaviour. Nilsson and Östlund-Nilsson^10^ also provide evidence of widespread hypoxia tolerance in tropical reef fish, but the critical O_2_ limit of clownfish was not examined in their study nor do these fish necessarily associate with the sources of hypoxia suggested by these authors (i.e. within branching corals or in tidal lagoons closed off to water exchange). Therefore, understanding the nature of O_2_ change around the surface of sea anemones and the level of hypoxia tolerance shown by *Amphiprion* species has the potential to provide extra insight into the factors leading to the evolution of what Nilsson and Östlund-Nilsson^10^ describe as “widespread hypoxia tolerance in coral reef fishes”.

## Methods

### General fish and sea anemone holding and acclimation

Forty nine (49) clownfish (*A. frenatus*) (mean ± SE; mass = 5.8 ± 0.4 g; length = 6.0 ± 0.1 cm) were maintained within the MAREE (MARine Experimental Ecology) recirculation facility at the Leibniz-Center for Tropical Marine Ecology (ZMT, Bremen); all 49 fish were used in the experiments described below. Fish were originally from Indonesia but were the third generation of a sizeable ZMT breeding stock and were held for at least nine months prior to experimentation. Fish were provided sea anemones (*Entacmaea quadricolor*) and terra cotta flowerpots for shelter and they were fed a mixed diet of *Mysis* and *Artemia* each day. Food was withheld on the day of experimentation. Eight anemones (*E. quadricolor*) from Indonesia were obtained from a commercial supplier, and varied between 12–15 cm diameter in size. The anemones were maintained at ZMT for several years before these experiments. Animal ethics approval was granted from Der Senator für Gesundheit (Title: Temperaturabhängigkeit von Korallenrifffischen) and all experiments were performed according to the relevant guidelines and regulations.

### Does hypoxia and/or hyperoxia exist among the sea anemone tentacles? O_2_ measures at 3 distances from the surface of *E. quadricolor* as a function of light intensity, with and without the presence of *A. frenatus*

O_2_ measuring experiments were performed on eight individual *E. quadricolor* housed in one of two 12.6 l tanks (30 cm long × 20 cm wide × 21 cm deep). These 2 measuring tanks were in turn housed in a single large holding tank (70 × 62 × 21 cm with seawater set to a depth of 16 cm (69 l). Two water pumps (Eheim 300, Eheim GmbH, Deizisau, Germany) in the main holding tank supplied the two inner measuring tanks with a continuous overflow of seawater at a rate of 2 l min^−1^. The main holding tank was aerated by an air pump (M2K3-WS3, Rebie, Germany) equipped with an air stone. The entire tank set up was enclosed by a framed light-proof canopy (120 cm high). UV sterilised seawater (Artificial seawater from Red Sea Salt crystals, Red Sea, Germany) was filtered to 0.2 µm (1500 cm^2^ capsule filter, Pall Corporation, USA) and supplied to the tank setup each day, after which water temp was controlled to 25 ± 0.5 °C as a result of the experiment being performed in a temperature controlled room with the additional assistance of an aquarium heater (TetraTec HT-300, Tetra, Melle, Germany).

Two robust fibre optic O_2_ dipping probes (OXROB10, Pyro Science GmbH, Aachen, Germany) were “sleeved” vertically in two hollow 20 cm long plastic shafts (OD = 6 mm, ID = 4 mm) that possessed a 6 cm long cylindrical plastic mesh “cage” at the bottom end. The principle of this technique was that a laboratory clamp and stand set up would position the mesh cage vertically on the surface of the anemone so that the O_2_ dipping could be moved up and down within the sleeve and that the probe tip could be positioned at 3 set distances from the central region of the anemone surface: 0.2 cm (i.e. just above the surface of the oral disk), 1.2 cm and 2.2 cm. The final 2.2 cm distance typically corresponded to a level around the top of the anemone’s tentacles. The mesh cage was designed to protect the probe tip from damage and also keep anemone tentacles clear of the probe, allowing free movement of water across the probe tip. Because the anemones sometimes retracted and expanded their tentacles, or even physically moved around the measurement tanks, the position of the dipping probe was monitored continuously and moved accordingly, to ensure that the correct measurement height was maintained at all times, and that the probe was positioned in the centre (vs. edge regions) above the anemone. O_2_ readings from the two dipping probes were measured and recorded using a Firesting 2 channel O_2_ meter (Pyro Science GmbH, Aachen, Germany) connected to a PC. Water temperature in the main holding tank was also monitored using a submersible TSB36 temperature sensor (Pyro Science GmbH, Aachen, Germany) connected to the 2 channel Firesting. Under dark conditions or when a clownfish was present, a low light sensitive CCD camera (QC3448, B&W, Taiwan) was used to check the position of the dipping probes relative to the anemone. Supplementary light from an infra-red light source (IR-12/65LED, Monacor GmbH, Bremen, Germany) assisted observations during dark measuring periods.

An LED aquarium lighting system (Aqua Illumination, model Hydra FiftyTwo HD, Iowa, USA) was mounted to the inside roof of the canopy frame, so that light intensity (measured as photon flux density, PFD) at the surface of the measuring tanks was either 0 µmol m^−2^ s^−1^ (lighting off and canopy fully covered), 55 µmol m^−2^ s^−1^ or 110 µmol m^−2^ s^−1^. Regardless of PFD, lighting spectra from the various LEDs in the Aqua Illumination module was fixed to provide a primary peak in wavelength around 475 nm (blue) and a smaller secondary peak around 680 nm (red). This spectral profile was the same across all PFDs and was selected on the basis of it being optimal for photosynthesis^13^. The three PFDs were calibrated and confirmed using a quantum sensor (LI-192SA, LI-COR, Nebraska, USA) connected to a portable meter (LI-250A, LI-COR, Nebraska, USA). The Aqua Illumination module was set to provide a 12 L:12D light cycle with a 1 h ramp up and down for dawn (0600 h) and dusk (1800 h).

To ensure that individual anemones were stress-free and not retracted on the day of experimentation, they were acclimatised overnight to the measuring tank for a period of >12 h. The measurement tank was not initially placed in the experimental holding tank (above), but was instead placed in a separate MAREE tank and received a flow of oxygenated MAREE seawater at 25 °C. On day 1 of the experiment, the entire measuring tank and anemone were quickly transferred within 1 min to the experimental holding tank (see above). The water lines running from the pump in the holding tank were then moved into the measurement tank and the anemone was left to acclimatise to any potential disturbance for a further 1 h period. All of the examined anemones were expanded and behaved normally at this point. For each experiment, light in the experimental tank was randomly assigned to either 55 or 110 µmol m^−2^ s^−1^ from the outset, and anemones were left undisturbed for 20 mins to acclimatise to this level of light, after which a 30 min measure of O_2_ at each of the 3 distances (0.2, 1.2 and 2.2 cm) was made consecutively. After the 2.2 cm recording was made, the PFD level was changed to the next random level and the same measurement procedure was followed as above. Once all O_2_ measures at 55 or 110 µmol m^−2^ s^−1^ were complete, the Aqua Illumination lighting system was turned off and the experiment was run at 0 µmol m^−2^ s^−1^ with the canopy covers down to ensure the experiment took place in total darkness and that photosynthesis could not occur. The positioning of the O_2_ dipping probe relative to the anemone was verified using images from the CCD camera on a monitor screen, and with manual adjustments of the mesh cage on the anemone surface. Aside from light from the monitor screen, only a low level of background yellow light was supplied to the temperature controlled room, and 0 µmol m^−2^ s^−1^ was recorded by the LI-COR meter above the measurement tank.

At the end of Day 1, a single clownfish (*A. frenatus*) was placed in each of the two measuring tanks for overnight acclimation. These fish immediately took refuge in the anemones. At the start of Day 2, the holding tank was given a complete exchange of seawater (which did not require any disturbance of water level or flow in the measurement tank), and this ensured that water quality was maintained. Thereafter, the O_2_ measuring protocol from Day 1 (as above) was repeated to assess O_2_ levels in the anemone, but on this occasion with *A. frenatus* present in the anemone. All of the clownfish tested associated strongly with the anemone.

For each distance above the anemone, a 2-way repeated measures ANOVA was used to test the effects of lighting level and fish presence on anemone O_2_ levels. Normality and variances were tested using a Shapiro-Wilk and Brown-Forsyth test, respectively. In the event of significant main effects or an interaction being detected, a Holm-Sidak method was used to make specific *post-hoc* comparisons.

### Resolving the hypoxia tolerance of *A. frenatus*: Are clownfish able to physiologically tolerate the O_2_ conditions observed in anemones?

Closed intermittent-flow respirometry was used to measure the mass-specific rate of O_2_ consumption ($\dot{M}{O}_{2}$, mg O_2_ g^−1^ h^−1^) of 41 *A. frenatus* for the purpose of resolving the hypoxia tolerance level of this species. The $\dot{M}{O}_{2}$ of individual fish was measured in a Perspex respirometry chamber that contained a magnetic stir bar (length 24 mm × 3 mm wide) positioned within a circular 40 mm wide × 10 mm deep recess in the base of the chamber. The recessed stir bar provided internal mixing during closed respirometry and was partitioned away from fish by a perforated plate. Different sized chambers were available for respirometry (92.5–160 ml) and selection was based on chamber volume being 20–30 × the volume of fish as recommended by Clark *et al*.^14^. Only two respirometer chambers were in operation at any given time and these were housed in a 70 × 62 × 21 cm reservoir tank with seawater set to a depth of 18 cm (78 l). For each experimental run, the reservoir tank was held in a temperature controlled room at 25 ± 0.5 °C and was filled with UV sterilized seawater filtered to 0.2 µm (1500 cm^2^ capsule filter, Pall Corporation, USA). The reservoir tank was supported on wooden blocks above a magnetic stir plate (Variomag Telesystem, Germany) which controlled the rotational spin of the stir bar in the respirometer chamber. A small 2.5 l min^−1^ pump (Mi Mouse, Sicce, Pozoleonne, Italy), connected to a portable PC notebook via a relay control unit system (USB Net Power 8800 Pro, Aviosys, Taiwan), was used to flush aerated water from the reservoir through the respirometer chamber. Once fish were sealed in the respirometry chamber, a repeating cycle of flush (1 min), wait (0.5 min) and measure (4–8 min) was initiated by customised software on the PC. Due to differences in fish size, the duration of the measurement phase was variable among fish (i.e. 4–8 min) to ensure a 5–10% drop in O_2_ over the measurement phase. The linear decline in the chamber oxygen during the sealed measurement phase was recorded by a contactless oxygen sensor spot system (OXSP5, Pyro Science GmbH, Aachen, Germany) connected to a 2-channel Firesting oxygen meter (Pyro Science GmbH, Aachen, Germany) and PC. $\dot{M}{O}_{2}$ was subsequently calculated using the methodology of Schurmann and Steffensen^15^. Bacterial respiration was accounted for by removing fish from the chambers at the end of experimentation, measuring the background O_2_ consumption of empty chambers and subtracting background $\dot{M}{O}_{2}$ from fish $\dot{M}{O}_{2}$ ^16^. The respirometer setup was screened off with white lining material to minimise external disturbance. To exclude the effects of specific dynamic action (SDA) on *M*O_2_^17^ all clownfish were starved for at least 24 h and then carefully introduced to the respirometer chamber around 17:00 h on the first day of experimentation. The reservoir tank was fully aerated with an air stone connected to an air pump, unless a hypoxia experiment was underway (see below). The whole respirometry system was dismantled after each experiment and scrubbed with ethanol to preclude bacterial respiration in subsequent runs.

After individual fish were introduced to the respirometers on Day 1, $\dot{M}{O}_{2}$ was measured repeatedly overnight and part way into the next day until >120 $\dot{M}{O}_{2}$measures were obtained. At that point, the standard metabolic rate (SMR. i.e. the best estimate of basal metabolism under experimental conditions) of fish under fully oxygenated conditions was estimated by plotting the frequency distribution of $\dot{M}{O}_{2}$ data and extracting the 15^th^ percentile value according to the methodology of^18–21^. Once SMR was resolved, the % air saturation level of seawater within the reservoir tank was reduced in progressive steps by bubbling nitrogen gas through seawater instead of air. Two water pumps (Eheim 300, Eheim GmbH, Deizisau, Germany) were used to mix the reservoir tank. The target O_2_ levels in the reservoir tank were adjusted to ensure that the measured O_2_ level in the middle of the closed respirometry cycles progressed in steps across the following 6 levels: 60, 40, 30, 25, 20, 15, 10 and 5% air saturation. $\dot{M}{O}_{2}$ was measured 3 times at 60, 40, 30 and 25% air saturation, twice at 20% air saturation and once at 15, 10 and 5% air saturation. The critical O_2_ saturation (*S*_crit_) limit of *A. frenatus* was subsequently measured using the methodology of Behrens and Steffensen^22^ and Cumming and Herbert^23^. In brief, mean $\dot{M}{O}_{2}$ at each O_2_ level was calculated and a repeated measures one-way ANOVA used to test the null hypothesis that O_2_-specific values of $\dot{M}{O}_{2}$ were not significantly below the measure of SMR under fully oxygenated conditions. A Holm-Sidak *post-hoc* test identified $\dot{M}{O}_{2}$ values that failed this hypothesis, which signified that fish could no longer regulate SMR at those O_2_ levels. A linear regression was subsequently plotted through that $\dot{M}{O}_{2}$ data with a forced y intercept of zero. SMR was extrapolated across the entire range of O_2_ and the associated point of intercept between the two linear equations identified *S*_crit_ in units of % air saturation. O_2_ levels >80% air saturation were considered as being fully air-saturated (i.e. normoxic).

To satisfy the assumption that control SMR can be extrapolated across the full range of O_2_^22^, and to ensure that *A. frenatus* was a strict O_2_ regulator (at least at O_2_ levels >20% air saturation), it was necessary to check whether the SMR of *A. frenatus* was indeed constant under reduced O_2_ conditions. The $\dot{M}{O}_{2}$ of individual clownfish was therefore screened in the same respirometry system as above. However, after an initial 1 h settlement phase in fully air-saturated seawater (normoxia), fish $\dot{M}{O}_{2}$ was measured at one of four levels of reduced O_2_ (60, 40, 30 and 20% air saturation) over an approximate period of 15 hours (i.e. typically overnight). A customised O_2_ control unit (ZMT EY design, Bremen, Germany) was used to hold O_2_ levels steady in the reservoir tank. Within this setup, a galvanic O_2_ probe (CellOx, WTW, Germany) connected to an oxygen meter (Oxi 3310, WTW, Germany) monitored the O_2_ saturation level of the reservoir tank and communicated the O_2_ level to a portable PC via USB connection. Customised software then manipulated tank O_2_ around the desired set point by allowing the flow of nitrogen gas (for deoxygenation) or air (for oxygenation) from a solenoid gas valve as required. O_2_ setpoints on the controller were set to ensure that fish within the measuring phase of respirometry were exposed to an average level of 60, 40, 30 or 20% O_2_ saturation. At least 120 $\dot{M}{O}_{2}$ measures were obtained from each fish at each O_2_ level and SMR was calculated as above. Following a Shapiro-Wilk and Browne-Forsythe test for normality and equal variances, respectively, a one-way ANOVA was used to test the effect of O_2_ on SMR and, where appropriate, the Holm-Sidak method was used to test whether the fully oxygenated (i.e. control) SMR value was different to SMR under reduced O_2_ levels.

Significance in all statistical tests was accepted at P < 0.05 and tests were performed using SigmaPlot version 13.0.

## Results

### O_2_ levels above the sea anemone *E. quadricolor*

**Figure 1:**
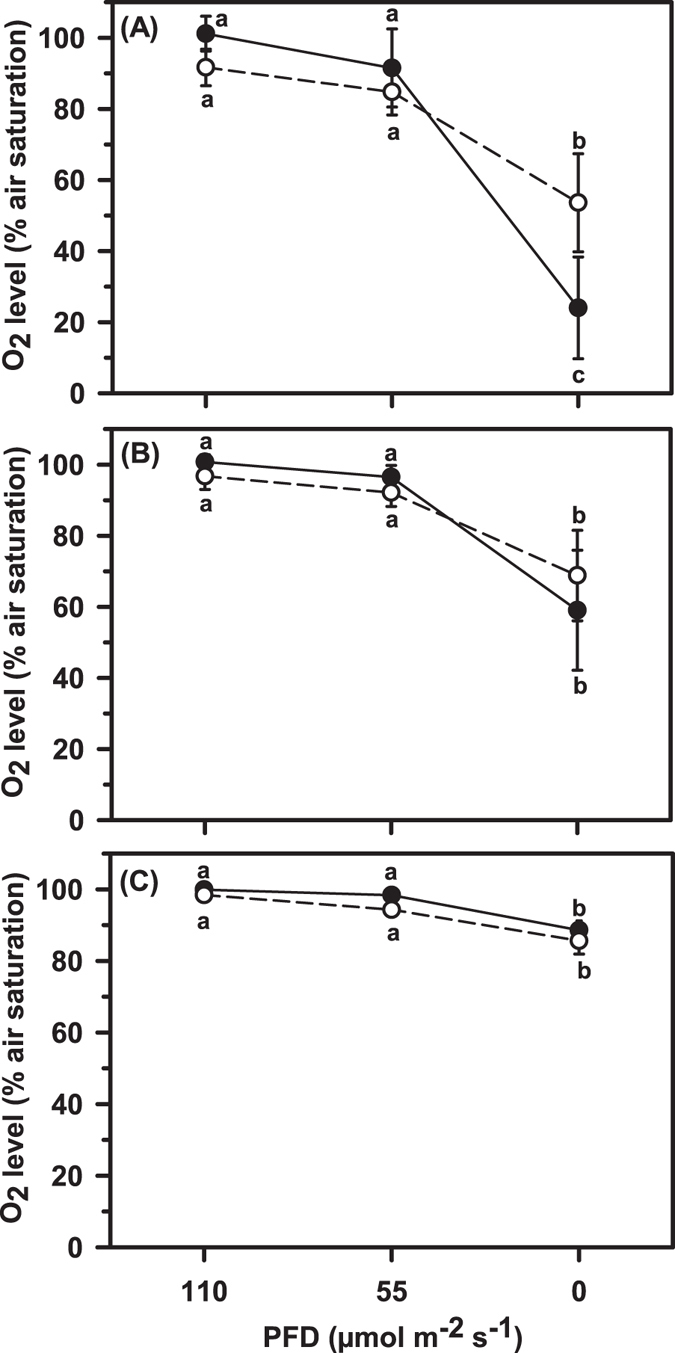
Oxygen levels (% air saturation) measured at a distance of (**A**) 0.2 cm, (**B**) 1.2 cm and (**C**) 2.2 cm above the surface of *E. quadricolor*, either in the absence of *A. frenatus* (closed symbol and solid line) or with *A. frenatus* present (open symbol and dashed line), at 3 different light intensity levels (PFD = 0, 55 and 110 µmol m^−2^ s^−1^). Data are average values ± 95% CI. Dissimilar letters indicate a significant difference (P < 0.05). N = 8 anemone and 8 clownfish (i.e. 8 separate pairings) using a repeated measures approach across the 3 distances and 3 light intensity levels.

Light intensity had a strong positive effect on dissolved O_2_ levels measured at a distance of 0.2 cm (2-way RM ANOVA. F_1,2_ = 55.26, P < 0.01. Fig. 1A), 1.2 cm (F_1,2_ = 25.73, P < 0.01. Fig. 1B) and 2.2 cm (F_1,2_ = 17.35, P < 0.01. Fig. 1C) from the anemone surface. In terms of specific O_2_ differences as a result of light intensity, average O_2_ levels were always >85% O_2_ saturated at 55 and 110 µmol m^−2^ s^−1^ and not significantly different (Fig. 1A–C. P > 0.05). However, O_2_ saturation levels at 0 µmol m^−2^ s^−1^ were always significantly less than the O_2_ saturation levels measured at 55 and 110 µmol m^−2^ s^−1^ and this trend was maintained across all distance measures (i.e. 0.2, 1.2 and 2.2 cm. P > 0.05) (Fig. 1A–C). Lower O_2_ levels were therefore always observed near the anemone under dark conditions.

At a light level of 110 µmol m^−2^ s^−1^ O_2_ levels were all generally >90% O_2_ saturated and there was no overall difference in O_2_ across the three measurement distances (i.e. O_2_ saturation at 0.2 cm = O_2_ at 1.2 cm = O_2_ at 2.2 cm)(F_1,2_ = 2.88, P > 0.05) (Fig. 1A–C). In contrast, at 55 µmol m^−2^ s^−1^, O_2_ levels increased with distance from the anemone surface (F_1,2_ = 8.49, P < 0.01) (Fig. 1A–C), because O_2_ was significantly lower close to the anemone surface (88% O_2_ saturated at 0.2 cm) compared to measures further away at 1.2 and 2.2 cm (i.e. 94 and 96% O_2_ saturated respectively. P > 0.05). Measurement distance had an even more pronounced effect on O_2_ levels from the anemone surface at 0 µmol m^−2^ s^−1^ (F_1,2_ = 32.5, P < 0.01), because O_2_ progressively increased with distance (i.e. an average of 39% O_2_ saturation at 0.2 cm < 64% O_2_ at 1.2 cm < 87% O_2_ at 2.2 cm. P < 0.05).

O_2_ measures at 2.2 cm were all generally >85% O_2_ saturated, but the presence of *A. frenatus* reduced O_2_ levels very slightly (F_1,2_ = 5.96, P = 0.045. Fig. 1C). However, *post-hoc* comparisons did not corroborate this finding, because fish presence did not specifically adjust O_2_ levels at 2.2 cm under a light level of 110 µmol m^−2^ s^−1^ (P > 0.05), 55 µmol m^−2^ s^−1^ (P > 0.05) or 0 µmol m^−2^ s^−1^ (P > 0.05). The main effect of fish presence at 2.2 cm was not therefore considered to be biologically relevant as the difference was so small. Also at a distance of 1.2 cm the presence of fish had no influence over O_2_ saturation levels (F_1,2_ = 0.04, P > 0.05. Fig. 1B). However, in terms of O_2_ levels at a distance of 0.2 cm, a very strong interaction was detected between light intensity and the presence of clownfish (2 way RM ANOVA interaction. F_1,2_ = 23.58, P < 0.01). Specific *post-hoc* tests revealed that the presence of clownfish raised O_2_ significantly (from a severely hypoxic level), but only under dark conditions (P > 0.05. Fig. 1A).

### The hypoxia tolerance of *A. frenatus*

**Figure 2:**
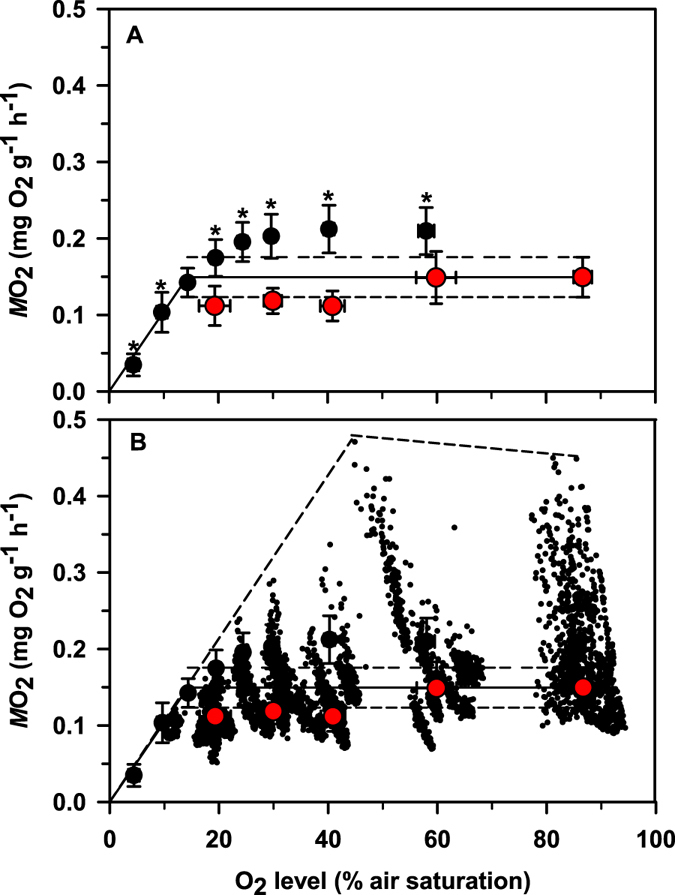
(**A**) The mass specific rate of oxygen consumption ($\dot{M}{O}_{2}$) of *A. frenatus* subject to O_2_ level changes (% air saturation) in seawater as resolved by static intermittent flow respirometry. Data symbols are mean ± 95% CIs and are coloured in black to show the $\dot{M}{O}_{2}$ response to an acute stepwise decline in O_2_ (N = 12) and are coloured in red to show SMR as a result of chronic O_2_ exposures (N = 29; 6–8 fish per chronic O_2_ treatment). The horizontal solid line shows an extrapolation of SMR from normoxic O_2_ levels (including horizontal dashed lines showing 95% confidence limits). The *S*_crit_ breaking point in SMR was calculated (see text for methodology) and found to occur at an O_2_ level of 14.3% air saturation. An asterisk indicates a significant increase or decrease from the normoxic SMR control value (P < 0.05). (**B**) This plot contains the same data as in (**A**) above, but individual data from the chronic O_2_ presentations are plotted to show the full range of measured $\dot{M}{O}_{2}$. Dashed lines surround the upper limit of measured $\dot{M}{O}_{2}$.

O_2_ level had a highly significant effect on the average $\dot{M}{O}_{2}$ of *A. frenatus* (1 way RM ANOVA. F = 105.01, P < 0.01) (Fig. 2A). In order to calculate *S*_crit_ (see methods), specific *post-hoc* tests identified that average $\dot{M}{O}_{2}$ was significantly lower than near-fully O_2_ saturated SMR at 5 and 10% air saturation (P < 0.05). Plotting a linear regression through these $\dot{M}{O}_{2}$ data points with a forced intercept of zero ($\dot{M}{O}_{2}$ = 0.0108 × O_2_ = 0.0046) and resolving the intercept between this regression and the extrapolated SMR value of 0.149 mg O_2_ g^−1^ h^−1^ revealed that the SMR of *A. frenatus* entered an oxy-conforming state below an *S*_crit_ value of 14.3% O_2_ saturation. However, because mean $\dot{M}{O}_{2}$ values were also significantly higher than normoxic SMR at 60, 40, 30, 25 and 20% air saturation (P > 0.05), it was necessary to check whether SMR does indeed remain steady during progressive hypoxia (at least until *S*_crit_) and that the SMR extrapolation technique of Behrens and Steffensen (2007) was appropriate for *A. frenatus*. Indeed, the higher average $\dot{M}{O}_{2}$ values observed could possibly be routine metabolism, not true resting SMR. Comparing the normoxic SMR of *A. frenatus* against the SMR of fish at 60, 40, 30 and 20% air saturation revealed that SMR was steady because there was no significant effect of O_2_ level on the SMR of *A. frenatus* (1 way ANOVA. F = 2.22, P > 0.05).

## Discussion

In similarity to the two banded anemone fish *Amphiprion bicinctus* in the study of Szczebak *et al*.^6^, *A. frenatus* was observed here to undertake aeration-like behaviour among the tentacles of host sea anemones during dark periods, but it is shown for the first time that this behaviour coincides with the development of low O_2_ (hypoxia) in the anemone (Fig. 1A). More importantly, it is demonstrated that the presence of fish performing aeration-like behaviours increases hypoxic oxygen levels to less physiologically challenging levels but only at a distance of 0.2 cm from the anemone surface. Aeration behaviour by coral reef fish among the branches of hard corals has been shown to increase O_2_ levels^7^, but the current data provides the first direct measure of fish manipulating O_2_ levels near a non-coral cnidarian (sea anemone) as a direct result of their behaviour. Due to the proximity of O_2_ change at the anemone surface and the high level of hypoxia tolerance shown by *A. frenatus*, we argue that the aeration behaviour of clownfish provides a direct O_2_ benefit only to the host anemone, not the fish. It should be noted, however, that this conclusion only applies to conditions of this study; it is likely that O_2_ graded with distance from the anemone would be very different in the wild where water exchange would fluctuate above and below 2 l min^−1^.

This study demonstrates for the first time that under well-lit conditions (i.e. 55 and 110 µmol m^−2^ s^−1^), seawater above a clownfish-hosting sea anemone was generally close to being fully air-saturated and was neither hypoxic or hyperoxic, nor was seawater O_2_ influenced by the presence of fish. Fv/Fm measures in the region of 0.62–0.69 from a PAM fluorometer indicated that zooxanthellae were photosynthesizing satisfactorily^13, 24^ within the anemone tentacles under the two lit conditions, but it is possible that the density of zooxanthellae was not sufficient to yield hyperoxic conditions above the anemone. Hyperoxia would ordinarily be expected if photosynthetic O_2_ production exceeds O_2_ consumption via respiration but this was not observed. A pronounced level of hyperoxia was seen at a distance of 2 mm above coral polyps^8, 9^ and this leads us to conclude that coral polyps probably have a higher level of photosynthetic O_2_ production, at least relative to respiratory O_2_ consumption, compared to the sea anemone *E. quadricolor*. With no evidence of hyperoxia above *E. quadricolor*, even at a distance of 0.2 cm, it is unlikely that this species would require extra ventilation to prevent any O_2_-related inhibition of photosynthesis during daylight hours. Whilst soft corals adopt a pulsating rhythm to vent excess O_2_ away from the tissue surface^12^, there appears to be no such mechanism in sea anemones, and the clownfish examined here were not observed to exhibit any form of ventilating behaviour when exposed to light at 55 and 110 µmol m^−2^ s^−1^. Hyperoxia probably isn’t therefore an important variable within the symbiotic association of anemone and clownfish.

Hyperoxia was not observed, but seawater of lowered O_2_ saturation was consistently recorded above the anemone under dark conditions and was deemed severely hypoxic at a distance of 0.2 cm above the anemone, at least in the absence of fish (Fig. 1A). This reduction in O_2_ under dark conditions likely represents the relative dominance of light independent respiration (O_2_ consumption) over light dependent photosynthesis (O_2_ production)^8, 9^. The magnitude of hypoxia 0.2 cm above *E. quadricolor* under dark conditions is consistent with that seen at 0.2 cm above coral polyps^8, 9^, but hypoxia in the anemone may also have been influenced by the partial closure, or “cupping”, of the flared oral disc under darkened conditions as this may have disturbed water flow and led to variable levels of water and O_2_ exchange around the tentacles.

The current results are comparable to the observations of Goldshmid *et al*.^7^ in that fish presence and their aeration-like behaviour increases O_2_ levels around the host and therefore eliminates any sign of hypoxia. Our observations are however the first showing the modulation of O_2_ as a result of clownfish behaviour in a *non-coral* host species. Our results also provide further detail in that clownfish exhibited aeration-like behaviour only under dark conditions and this behaviour appeared to raise O_2_ from a severely hypoxic level (<50% air saturation) to a less physiologically challenging level of oxygenation (>50% air saturation) only at a distance of 0.2 cm from the anemone surface. With evidence of clownfish not improving O_2_ conditions at a distances ≥1.2 cm it appears that fish increase O_2_ at only a highly localised scale above the anemone and therefore provide only a one-sided benefit to the anemone (see below for further discussion). This study is focussed on the clownfish-anemone association, but the same one-way benefit may also apply to branching corals, because low O_2_ also appears to persist at only a highly localised scale around the boundary layer of hermatypic corals^8, 9^ and damselfish are shown to reduce the severity of hypoxia within that boundary layer^7^.

With no information regarding the hypoxia tolerance of clownfish, respirometry was specifically performed to assess whether *A. frenatus* could tolerate the O_2_ levels observed in their anemone host. With surprisingly high levels of $\dot{M}{O}_{2}$ recorded during the progressive drop in O_2_ there was some initial uncertainty as to whether the SMR of *A. frenatus* was fixed and stable before it reached *S*_crit_ and therefore whether it was appropriate to resolve *S*_crit_ using the SMR extrapolation technique of Behrens and Steffensen^22^. Investigatory measures of SMR at 4 extra set levels of low O_2_ (20, 30, 40 and 60% air saturation) were therefore made and, with evidence of stable SMR (Fig. 2A), the SMR extrapolation technique of Behrens and Steffensen^22^ was validated. By taking care to ensure SMR was recorded (*vs*. routine $\dot{M}{O}_{2}$ that incorporates a component of spontaneous activity) an *S*_crit_ breaking point of only 14.3% O_2_ saturation was resolved. As the *S*_crit_ point of O_2_ conformity occurs at an extremely low level of oxygen, *A. frenatus* can therefore be described as an extremely hypoxia tolerant species and there is no doubt that it could tolerate the O_2_ levels observed in *E. quadricolor*. Even though the gills of *A. frenatus* would never be bathed in seawater layered 0.2 cm above *E. quadricolor* (the distance is simply too close to the anemone), this clownfish species could still theoretically tolerate and survive the severe hypoxic conditions seen at this distance in the dark (i.e. 24% air saturation). Moreover, the O_2_ levels seen at distances considered more relevant to the fish ( ≥ 1.2 cm) would not be deemed hypoxic (i.e. O_2_ > 50% air saturation) and, with a tolerant *S*_crit_ value of 14.3%, *A. frenatus* would probably retain a sizeable fraction of aerobic metabolic scope and would not be challenged physiologically at these O_2_ levels. Interestingly, examining the $\dot{M}{O}_{2}$ range from our SMR experiments shows that the maximal observed $\dot{M}{O}_{2}$ values were protected and not subject to reduction at O_2_ levels >48% air saturation (Fig. 2B) but whether the $\dot{M}{O}_{2}$ data range relates to true aerobic metabolic scope was never confirmed. The low *S*_crit_ threshold of *A. frenatus* is especially impressive given that the experiments were performed at 25 °C, a temperature expected to yield high metabolic rates at a time of low O_2_ solubility. In order to place the low *S*_crit_ value of 14.3% into context, Cook and Herbert^25^ used similar methodologies to establish the hypoxia tolerance threshold of a coastal sparid species from New Zealand (*Pagrus auratus*), but this species revealed a much higher *S*_crit_ value of ~28% air saturation at a considerably cooler temperature (18 °C). With the exception of one species, the 14.3% *S*_crit_ level of *A. frenatus* is also less than the *S*_crit_ of 32 species reported by Nilsson and Östlund-Nilsson^10^. Direct comparisons with this study are difficult, however, because Nilsson and Östlund-Nilsson^10^ performed their experiments at 30 °C, not 25 °C. It is also questionable whether fish resting $\dot{M}{O}_{2}$ (i.e. SMR) was truly measured in their study given the known errors associated with non-intermittent flow methodologies^26, 27^ and the lack of acclimation time provided to fish in the respirometers. In order to record *S*_crit_ according to true definitions, it is important to establish the break in true resting $\dot{M}{O}_{2}$ (i.e. SMR, resolved after a substantial time of rest and with no issue of respiratory or nitrogenous waste^14, 19, 22, 26, 27^). It is therefore possible that the true *S*_crit_ value of the 32 species in Nilsson and Östlund-Nilsson^10^ are even lower than the values they report and therefore comparable to *A. frenatus* in the current study under comparable temperatures^26^.

Hypoxia was restricted to the boundary-like layer of sea anemones in this study (Fig. 1A), and clownfish are extremely low O_2_ tolerant (Fig. 2), so based on the collective observations thus far, it is proposed that clownfish aerate the anemone with no apparent respiratory benefit to themselves. Our observation that clownfish use their tails to aerate their host anemones whilst poking their heads out from among the anemone tentacles lends support to this proposition, because no deoxygenation or O_2_ replenishment was seen at distances where the respiratory gill structures of the fish would be located. As clownfish are strict O_2_ regulators (and therefore preserve maintenance $\dot{M}{O}_{2}$ under progressive hypoxia to 14.3% air saturation. Figure 2), the aeration-like behaviour performed by *A. frenatus* would serve only to increase metabolic expenditure and would thus not likely provide fish with a metabolic benefit under the reasonably well-oxygenated conditions seen near host anemones (Fig. 1). Some species of fish that take refuge in a shelter appear to consume less oxygen and are therefore considered to have a metabolic advantage^28^, but shelter *per se* does not appear to adjust the O_2_ uptake, hence the metabolic energy expenditure of *Amphiprion* clownfish species^29^. It is not therefore obvious that *A. frenatus* receives any metabolic reward for performing the aeration-like behaviours described by Szczebak *et al*.^6^ (e.g. wiggling etc). Since the behaviour arguably conveys a metabolic cost to the fish, we agree with Szczebak *et al*.^6^ that these behaviours probably do contribute to the increased $\dot{M}{O}_{2}$ of clownfish-anemone pairings.

In contrast to clownfish, the benefits of aeration-like behaviour by fish are much more obvious for the anemone. Anemones are oxygen conformers^30, 31^ and therefore show a reduction in maintenance $\dot{M}{O}_{2}$ as O_2_ availability decreases, even at relatively high O_2_ saturations that would not be deemed hypoxic. Hypoxia, and even modest reductions in environmental O_2_, therefore pose a physiological challenge for anemones if their ecology demands that they must feed, grow and maintain core bodily functions. The fact that *A. frenatus* eliminates hypoxia 0.2 cm above the anemone in the dark would almost certainly convey a direct metabolic benefit to the anemone, because the resting $\dot{M}{O}_{2}$ (SMR) of anemones would not be subject to such a pronounced decrease during hypoxia. It is for this reason that Szczebak *et al*.^6^ are likely correct again in their conclusion that anemone $\dot{M}{O}_{2}$ is potentially augmented by the presence of fish performing aeration-like behaviours. Clownfish therefore provide a one-sided metabolic advantage to the anemone, but the benefits for the anemone probably do not stop there. Indeed, when anemones host clownfish they show improved rates of growth^32^ and reproduction^2, 32^, and this may be at least partially attributable to the improved level of oxygenation that clownfish provide (Fig. 1A), although the benefits of physical contact and stimulation cannot be ruled out.

It is argued above that clownfish provide aeration for the sole benefit of the anemone but, if fish do not experience hypoxia themselves and are not therefore stimulated to wriggle by low O_2_, what serves to trigger their behaviour? As anemone are O_2_ conformers^30, 31^ and experience an O_2_ debt reaction in response to only modest levels of deoxygenation^32^, it is feasible that some form of “metabolic alarm signal” is released by the anemone at night. Indeed, a chemical signal could feasibly stimulate the aeration-like behaviour of clownfish, which in turn replenishes O_2_ around the anemone boundary layer (Fig. 1A) to safeguard the anemone host against respiratory distress. A potential chemical signal has yet to be identified, but this is not an unreasonable theory, because clownfish use olfactory chemical cues to locate healthy anemones so chemical communication is already a well-established part of their symbiosis^33–37^. However, clownfish exhibit flow-modulating behaviours even when not co-housed with host anemones, albeit at lower rates^6^. As such, some aspects of this type of behaviour may be genetically-controlled and not require an environmental signal for elicitation. If clownfish are burdened with a metabolic cost of aeration and receive no respiratory benefit themselves, another interesting aspect to consider is whether the behaviour of clownfish would be considered more altruistic than symbiotic. This might seem true from a physiological point of view but, with all factors considered, the aeration behaviour of clownfish would probably not be considered entirely altruistic, because these fish rely on anemones for safe shelter, food (nutrients) and reproduction (egg laying)^1, 4^ and are therefore rewarded via alternate pathways over the long term. It is common for some anemonefish to remain within a few inches of their host for their entire lifetime^38^.

Without any evidence of widespread hypoxia among the tentacles of *E. quadricolor* to date, it is not yet known why *A. frenatus* appear to be so physiologically tolerant of low O_2_. A low *S*_crit_ is commonly considered an indicator of hypoxia tolerance, but one possibility to consider is it might not actually represent an evolved response to hypoxic pressure. A low *S*_crit_ could simply be the indirect consequence of high metabolic capacity and actually have no functional relevance to hypoxia tolerance. For example, Cook *et al*.^19^ demonstrated that an experimental reduction of the maximal metabolic rate ($\dot{M}{O}_{2}$_*max*_) and the aerobic metabolic scope (AMS) of a temperate sparid fish yielded a pronounced increase in the *S*_crit_ despite no change in the SMR or any difference in the historical O_2_ environment of their fish. As *S*_crit_ reflects the point at which $\dot{M}{O}_{2}$_*max*_ is equal to SMR and AMS is zero^19, 39^, the low *S*_crit_ of clownfish and of other tropical reef fishes^10^ could theoretically be the result of fish having an enlarged maximum rate of $\dot{M}{O}_{2}$ ($\dot{M}{O}_{2}$_*max*_ *)* and an enlarged aerobic metabolic scope (AMS) that simply supports ecologically-relevant activity, such as reproduction, anti-predatory defence etc, under non-hypoxic conditions. However, this seems unlikely since $\dot{M}{O}_{2}$_*max*_ and hypoxia tolerance are inversely correlated in tropical damselfish^40^ (Nilsson *et al*.^40^) and the maximal observed $\dot{M}{O}_{2}$ of *A. frenatus* would not be considered as being especially large for its body size (Fig. 2B).

So why do *A. frenatus* possess such a low *S*_crit_ value? Extreme low O_2_ that challenges clownfish physiologically has yet to be seen in anemones (Fig. 1), but that doesn’t necessarily mean it doesn’t exist. Although not observed in our relatively short term experiment, there remains the possibility that extreme widespread hypoxia could develop more strongly over time in a deeply-cupped, contracted sea anemone at night and that extreme hypoxia tolerance is required for fish to sleep under these conditions. Indeed, using O_2_ probes at non-standardised distances during pilot experimentation revealed values <50% O_2_ saturation in several anemones for at least one third of the night. In some cases, values as low as 30% O_2_ saturation were observed in cupped anemones (Kunzmann, A. and Petereit, J. Personal observation). In this scenario, a low *S*_crit_ would effectively safeguard the SMR of sleeping clownfish and there would probably be selective pressure for extreme low O_2_ tolerance under these conditions. Low O_2_ appears to develop slowly over a night time period in coral branches^11^ and there is evidence that fish sleep, and even provide aeration, under these conditions^7^. Also, anemones are known to reside on very shallow reefs and within reef lagoons^41^, where oxygen saturation might be expected to drop. However, unfortunately there are no data comparing the behaviour of clownfish against the temporal dynamics of O_2_ in anemones, so future investigations into this issue will likely provide important insights.

In conclusion, sea anemones experience severe hypoxia but only under dark conditions and within their surface boundary layer. Since clownfish ventilate their gills with water above this boundary layer, it is unlikely that *A. frenatus* experienced hypoxia under the conditions of the current study, yet they still undertook metabolically-expensive behaviours to aerate the anemone. Whilst this apparently selfless form of behaviour could be viewed as altruism, it is probably more reasonable that clownfish capitalise on the protective benefits of anemone-clownfish association (symbiosis) by ensuring the metabolic health of the host over the long-term. Whilst not a feature of the current study, more research is required to resolve whether clownfish ever experience hypoxia (e.g. whilst sleeping within a deeply cupped anemone at night) as this might potentially explain why these fish are so hypoxia tolerant.
